# Ferroptosis-related prognostic model of mantle cell lymphoma

**DOI:** 10.1515/med-2024-1090

**Published:** 2024-11-20

**Authors:** Qianwen Gao, Xin Wang, Yue Zhang, Jingjing Wen, Fangfang Wang, Zhimei Lin, Yu Feng, Jingcao Huang, Qian Li, Hongmei Luo, Xiang Liu, Xinyu Zhai, Linfeng Li, Siyao He, Ziyue Mi, Li Zhang, Ting Niu, Caigang Xu, Yuhuan Zheng

**Affiliations:** Department of Biology, School of Life Science, Sichuan University, Chengdu, China; Department of Hematology, West China Hospital, Sichuan University, Chengdu, 610041, China; Department of Hematology, Mian-yang Central Hospital, Mianyang, China; Department of Hematology, The Affiliated Hospital of Chengdu University, Chengdu, China; Department of Hematology, West China Hospital, Sichuan University, #37 Guo Xue Xiang Street, Chengdu, 610041, China

**Keywords:** mantle cell lymphoma, prognosis, immune microenvironment, bioinformatics, GEO

## Abstract

**Background:**

Mantle cell lymphoma (MCL) is a B-cell non-Hodgkin’s lymphoma. Ferroptosis, an iron-dependent programmed cell death, is closely related to cancer prognosis. In this study, we established a model of ferroptosis related genes for prognostic evaluation of patients with MCL.

**Methods:**

Using the single-cell RNA sequencing datasets GSE184031 and mRNA sequencing data GSE32018 from the Gene Expression Omnibus, we identified 139 ferroptosis-related genes in MCL. Next a prognostic model was constructed by Cox regression and Least absolute selection and shrinkage Operator regression analysis. Finally, we used CIBERSORT to analyze the immune microenvironment and the “oncoPredict” package to predict potential drugs.

**Results:**

In our model, the prognosis of MCL patients was assessed by risk scoring using 7 genes ANXA1, IL1B, YBX1, CCND1, MS4A1, MFHAS1, and RILPL2. The patients were divided into high-risk and low-risk groups based on our model, and the high-risk patients had inferior overall survival. Finally, according to our model and computational drug sensitivity analysis, four small molecule compounds, BMS-754807, SB216763, Doramapimod, and Trametinib, were identified as potential therapeutic agents for patients with MCL.

**Conclusion:**

In summary, we provide a prognostic model with ferroptosis-related gene signature for MCL. This study provides a prognostic model with ferroptosis-related gene signature for MCL. The results show that the model helps predict prognosis in MCL.

## 1 Introduction

Mantle cell lymphoma (MCL), a B-cell non-Hodgkin’s lymphoma, is characterized by its highly aggressive nature, resulting in a median overall survival (OS) of approximately 4.5 years following diagnosis [1]. According to the MCL International Prognostic Index classification, patients with low-risk, intermediate-risk, and high-risk disease exhibit OS rates of 83, 63, and 34%, respectively [2]. In general, newly diagnosed MCL patients have good response to chemotherapy and autologous stem cell transplantation [3]. The overall response rate to the first-line regimens for the patients are 60–97% [4]. The occurrence of drug resistance often leads to relapse in patients with MCL, resulting in a median survival rate of only 1–2 years. Furthermore, the majority of MCL cases are diagnosed at an advanced stage which significantly impacts long-term survival [5].

Ferroptosis, an iron-dependent form of programmed cell death, is characterized by accumulation of reactive oxygen species (ROS)-induced lipid peroxides that lead to cell membrane damage and cell death [6]. The initiation of ferroptosis can be attributed to two distinct cellular signaling pathways. First, aberrations in glutathione peroxidase 4 (GPX4) impair the antioxidant capacity of cells and promote ferroptosis processes. Second, disruption of intracellular iron homeostasis also triggers ferroptosis [7]. Previous research works have demonstrated the critical roles of ferroptosis in various types of lymphoma. For example, a prognostic model based on ferroptosis was established for diffuse large B cell lymphoma (DLBCL) [8]. And drugs targeting ferroptosis inhibited tumor growth in DLBCL [9]. Furthermore, Lepelletier et al. discovered that alterations of iron levels triggered apoptosis in MCL [10], a finding that suggested the potential of ferroptosis-targeting therapy in MCL. Therefore, we investigated the correlation of ferroptosis-related genes’ expression and prognosis of MCL. The aim of our study was to use bioinformatics tools to identify potential treatment targets in MCL ferroptosis cell signaling molecules.

## 2 Materials and methods

### 2.1 Single-cell sequencing data acquisition

Single-cell sequencing data and clinical information for four MCL samples were downloaded from the Gene Expression Omnibus (GEO, https://www.ncbi.nlm.nih.gov/geo/ [11]) database GSE184031. Samples from peripheral blood of MCL patients with CD19+ B cells were removed. We used the “Seurat” R package to create objects for data quality check. To exclude the effect of poor-quality cells, gene expression ranges from 200 to 2,500 were selected for retention, while erythroid cells and cells with >20% mitochondrial genes were excluded. Screening was done for the top 3,000 genes with the greatest differences in expression in all cells. Cells are then annotated and visualized based on the cell surface marker.

### 2.2 Transcriptome data acquisition

RNA sequencing data and corresponding clinical information of 24 MCL samples and 7 normal lymph node samples were obtained from GEO database GSE32018. The gene expression profiles and clinical information of GSE93291 and GSE42854 were downloaded as the training and validation sets, respectively.

### 2.3 Selection of ferroptosis-related genes

Nine marker genes of ferroptosis were obtained from the FerrDb database, these 9 genes indicate the occurrence of ferroptosis [12]. The percentage of ferroptosis marker genes in each cell was further calculated using the “PercentageFeatureSet,” and the cells were divided into high and low ferroptosis groups using the median as a cut-off. A total of 655 differentially expressed genes (DEGs) between high and low ferroptosis groups were obtained by “FindMarkers.”

### 2.4 Screening DEGs in MCL

The limma package was used in the GSE32018 dataset to look for differential genes between MCL patient samples and non-MCL samples, and *p* < 0.05 and |Log FC| > 0.8 were considered as DEGs.

### 2.5 Functional pathway enrichment analysis

Gene Ontology (GO), Kyoto Encyclopedia of Genes and Genomes Ontology (KEGG), and gene set enrichment analysis (GSEA) based on Molecular Signatures Database enrichment analysis were performed, respectively.

### 2.6 Generation of the prognostic model

The univariate Cox regression analysis was used to identify genes with prognostic value by the “survival” package. Least absolute selection and shrinkage Operator (LASSO) was used to identify candidate genes for risk score signature. Based on the model, risk scores may be calculated for each sample, allowing further classification of MCL patients into high and low-risk groups. GSE42854 and GSE132929 were selected as the validation dataset for the prognostic model. Risk scores were calculated for each sample, and the patients were divided into high and low-risk groups to compare the prognosis between the two groups.

### 2.7 Immune microenvironment analysis

Based on GSE93291 gene expression profiling, CIBERSORT was used to evaluate cellular composition in tissues from MCL patients [13]. The correlation of candidate genes and the immune microenvironment in prognostic models was performed by the “corrplot” package. We also used ESTIMATE package to predict the stromal score and immune score in patients with high-risk and low-risk MCL.

### 2.8 Drug sensitivity analysis

Sensitivity scores of each drug were calculated for patients in the high and low-risk groups using the “oncoPredict” package [14], and then 3D structure maps for each drug were obtained using PubChem (https://pubchem.ncbi.nlm.nih.gov/ [15]).

## 3 Results

### 3.1 Acquisition of ferroptosis-associated genes

**Figure 1:**
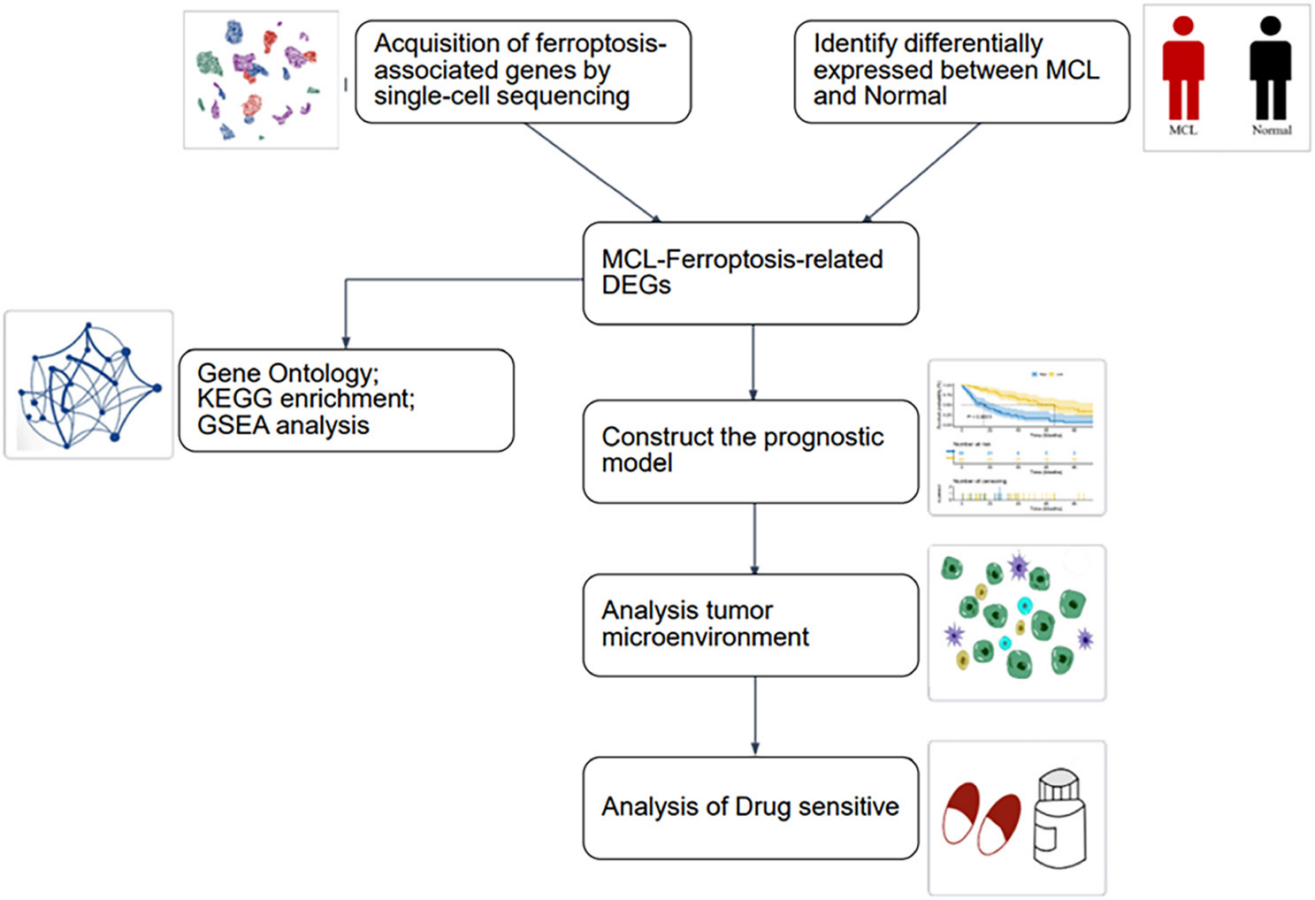
Diagram of work flow.

**Figure 2:**
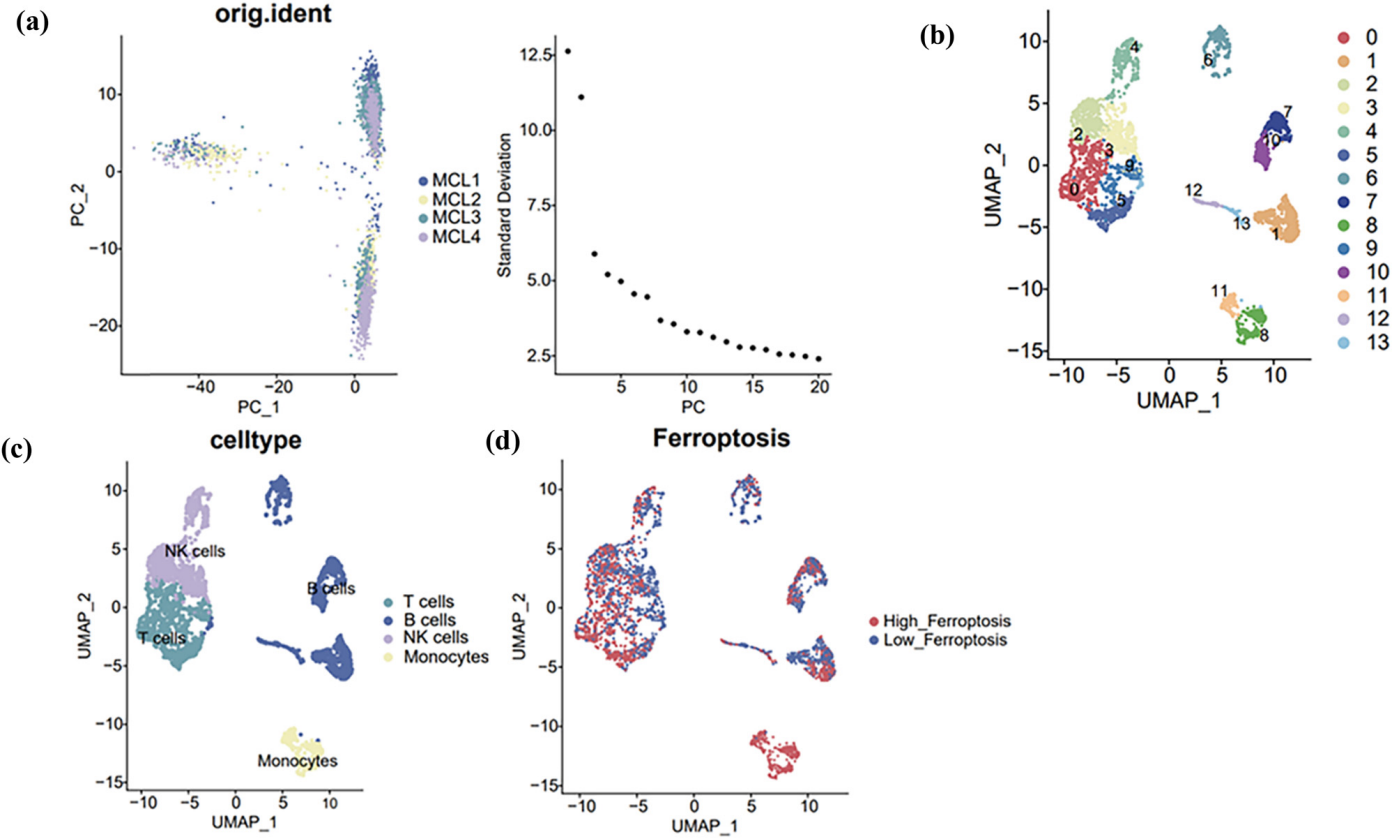
Single-cell sequencing analysis of MCL. (a) Good sample integration with no significant batch effects; (b) 13 clusters obtained after dimensionality reduction; (c) cell type annotations according to cell surface marker genes. The cells were annotated as T cells, B cells, NK cells, and monocytes; and (d) calculated the percentage of ferroptosis genes in each cell by “PercentageFeatureSet.” The cells were divided into high ferroptosis and low ferroptosis cells based on the median.

To generate prognostic model of MCL based on ferroptosis-related genes’ expression, we followed the workflow as described in Figure 1. Single-cell sequencing result of MCL patients were obtained from Gene Expression Omnibus GSE184031. After data integration and low-quality data depletion, as described in Section 2, the data exhibited well integration between samples and no significant batch effect (Figure 2a). After dimensional reduction, the cells were clustered into 13 clusters by the shared nearest neighbor and *k*-nearest neighbor algorithm (Figure 2b). Major immune-cell types, such as T cells, B cells, NK cells, and monocytes, were identified by cell surface marker genes (Figure 2c). Zhou and Bao established a FerrDb database by summarizing literatures related to iron death, and found nine genes indicating the occurrence of ferroptosis, namely, FTH1, GPX4, CHAC1, HSPB1, NFE2L2, PTGS2, SLC40A1, TF, and TFRC after screening [16]. We used these nine genes as ferroptosis marker gene and further analyzed the ferroptosis-related genes’ expression in those immune-cells. According to the expression values of these genes, the cells were divided into high-expression group of ferroptosis genes and a low-expression group with the median truncated (Figure 2d).

### 3.2 Identification of DEGs in MCL

**Figure 3:**
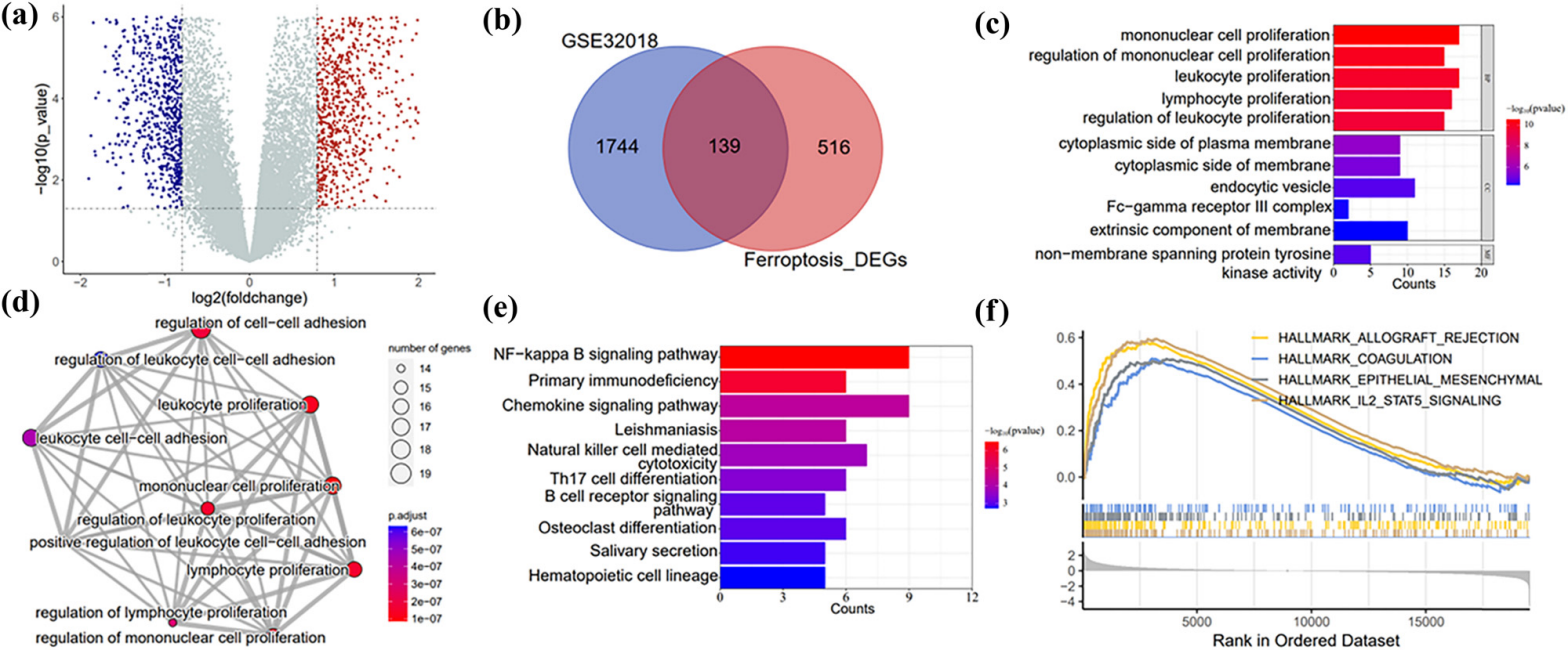
Differentially expressed ferroptosis-related genes and enrichment analysis. (a) Volcano map of DEGs in MCL patients and normal samples; (b) Venn diagrams of ferroptosis-associated genes and DEGs; (c) GO biological functions analysis; (d) interlinkage of GO enrichment analysis pathways; (e) KEGG pathway enrichment; and (f) enrichment plot of GSEA enrichment analysis.

Next we analyzed differentially regulated genes in MCL vs healthy tissue. As shown in the volcano plot in Figure 3a, a total of 1,883 DEGs were identified in MCL, compared with non-tumeric samples. A total of 1,005 genes’ expressions were upregulated in MCL, while 878 genes’ expressions were downregulated. Next we used the “PercentageFeatureSet” to obtain the percentage of ferroptosis marker gene in each cell, and separate the high ferroptosis cells from the low ferroptosis cells. Genes that differed between the two groups were considered ferroptosis-related gene. By cross-comparing DEGs with ferroptosis-related gene set, 139 differentially expressed, ferroptosis-related genes were identified in MCL (Figure 3b). Pathway enrichment analysis showed that the top three pathways in GO enrichment were mononuclear cell proliferation, regulation of mononuclear cell proliferation, and leukocyte proliferation (Figure 3c). The interconnections between the various pathways in the Biological Process subset of GO enrichment are shown in Figure 3d. In addition, KEGG enrichment analysis suggested that those 139 differentially expressed, ferroptosis-related genes were mainly involved in NF-κB signaling pathway, primary immunodeficiency pathway, chemokine signaling pathway, and so on (Figure 3e). At last, GSEA enrichment analysis showed that allograft rejection, coagulation, epithelial mesenchymal, and IL2-STAT5 signaling pathways were altered in MCL (Figure 3f). Overall, our results provided a landscape of ferroptosis-related signaling pathways in MCL.

### 3.3 Generation and validation of a prognostic model for MCL

**Figure 4:**
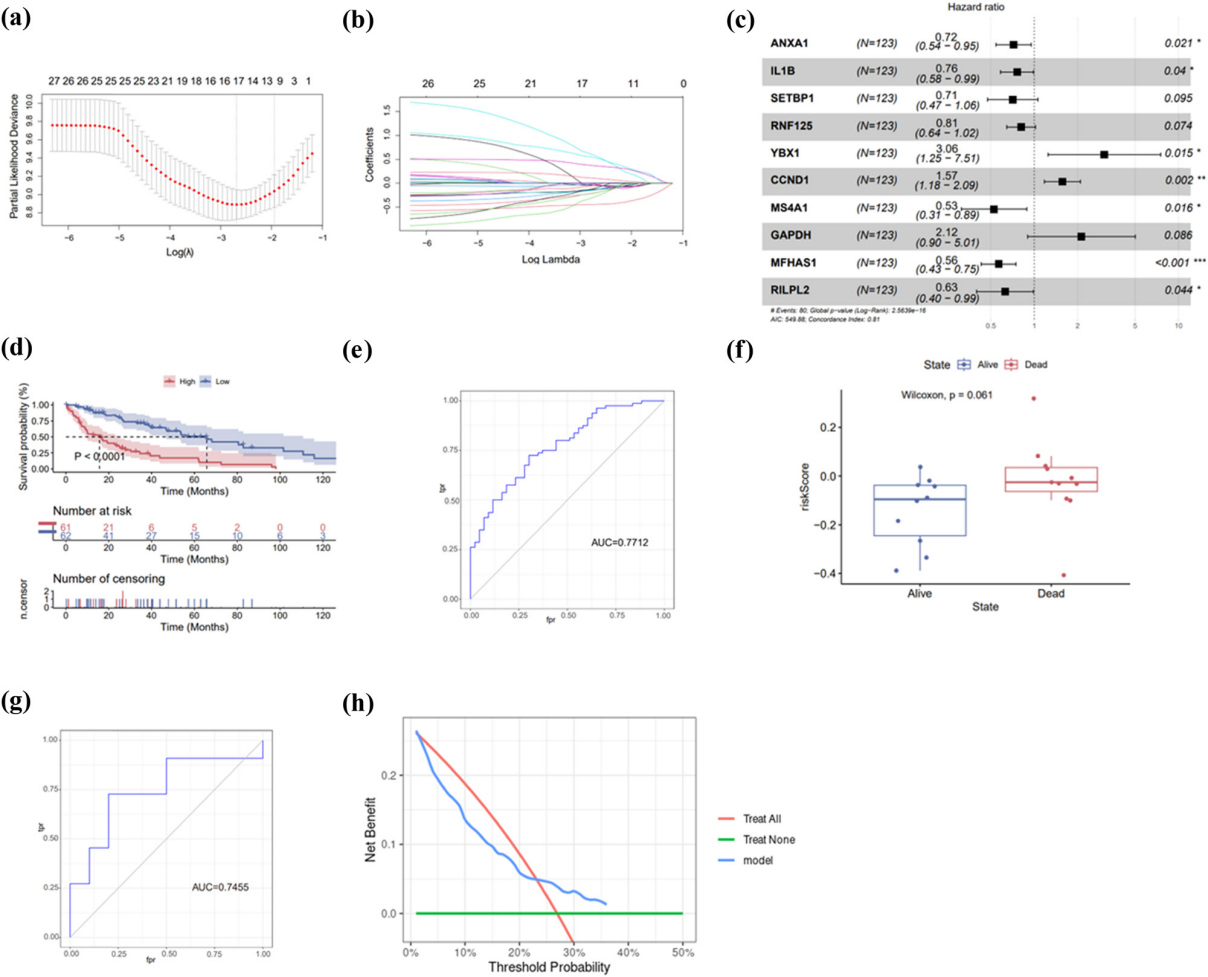
Generation and validation of a prognostic model. (a) and (b) Lasso regression of 17 candidate’s genes; (c) multivariate Cox regression analysis; (d) Kaplan Meier (KM) curve of GSE93291 in the high-risk and low-risk groups showed that high-risk patients had inferior OS; (e) ROC curves of training set GSE93291; (f) risk scores and survival of the validation set showed that patients with high-risk scores had worse survival than those with low-risk scores; (g) ROC curves of validation set GSE42854; and (h) decision curve analysis of model.

**Figure A1:**
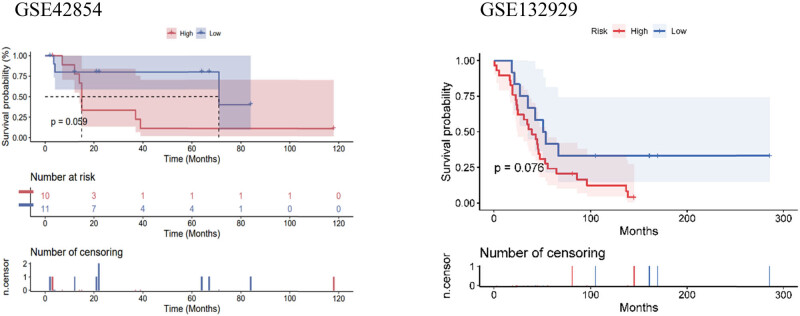
Kaplan Meier (KM) curve of GSE42854 and GSE132929.

We started with those 139 ferroptosis-related DEGs to generate a prognostic model for MCL. Univariate Cox regression analysis identified that the expression of 29 genes was each associated with OS in MCL with *p* < 0.05. Next we performed a LASSO regression analysis using the expression of those 29 genes. A total of 17 genes were identified as candidates as indicated in Figure 4a and b. At last, we performed a multivariate Cox regression and generated a prognostic model for MCL with seven genes’ expression. As shown in Figure 4c, the expression of seven genes, ANXA1, IL1B, YBX1, CCND1, MS4A1, MFHAS1, and RILPL2, could be used to calculate the risk score of MCL patients. Risk score = expression of ANXA1*(−0.3315964) + expression of IL1B*(−0.2757019) + expression of YBX1*(1.1179124) + expression of CCND1*(0.4495180) + expression of MS4A1*(−0.6428142) + expression of MFHAS1*(−0.5715639) + expression of RILPL2*(−0.4668621). In validation cohort GSE93291, we calculated the risk scores of each patient and divided the patients into high-risk vs low-risk groups based on the median risk scores. The patients in high-risk group had inferior OS, compared with the patients in low-risk group with *p* value less than 0.0001 (Figure 4d). The receiver operating characteristic (ROC) curve showed that the area under curve (AUC) of the model is 0.7712, a result indicating that the model provided good prediction of MCL prognosis (Figure 4e). To explore the accuracy of the model, we further used the validation set GSE42854. The clinical baseline table of dataset GSE42854 was performed in Table A1. As shown in Figure 4f, patients were lost because the disease had higher risk score than the alive patients, with an AUC of 0.745 (Figure 4g). Kaplan Meier (KM) curve analysis was also performed on the validation datasets GSE42854 and GSE132929, but there was no statistical significance (Figure A1).

## 4 Key genes’ expression in MCL immune cells

**Figure 5:**
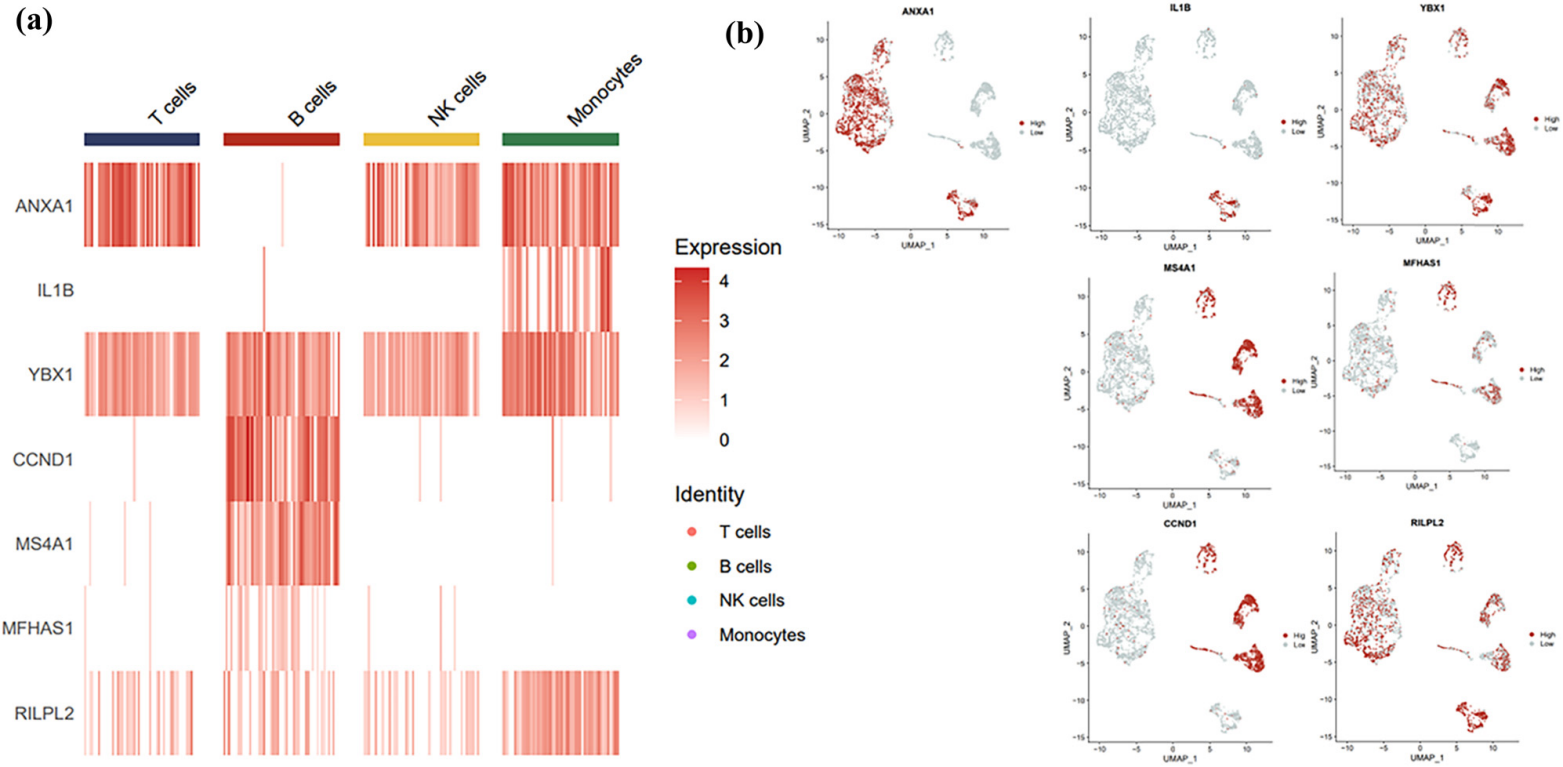
Locations of model genes. (a) and (b) Locations of seven genes in immune cells showed that YBX1 and RILPL2 were expressed in T cells, B cells, NK cells, and monocytes. CCND1, MS4A1, and MFHAS1 were mainly expressed in B cells. IL1B was expressed in monocytes. ANXA1 was primarily expressed in T cells, NK cells, and monocytes.

Of interest, we also investigated the seven-genes’ expressions in MCL tumor microenvironment. As shown in Figure 5a, those seven genes are differentially expressed in immune cells that infiltrated in MCL tumor bed. YBX1 and RILPL2 were highly expressed in all tested immune cells; while CCND1, MS4A1, and MFHAS1 were mainly expressed in B cells; IL1B was restrictively expressed in monocytes; and ANXA1 was expressed in T cells, NK cells, and monocytes, but not in B cells (Figure 5a and b).

### 4.1 Immune microenvironment analysis

**Figure 6:**
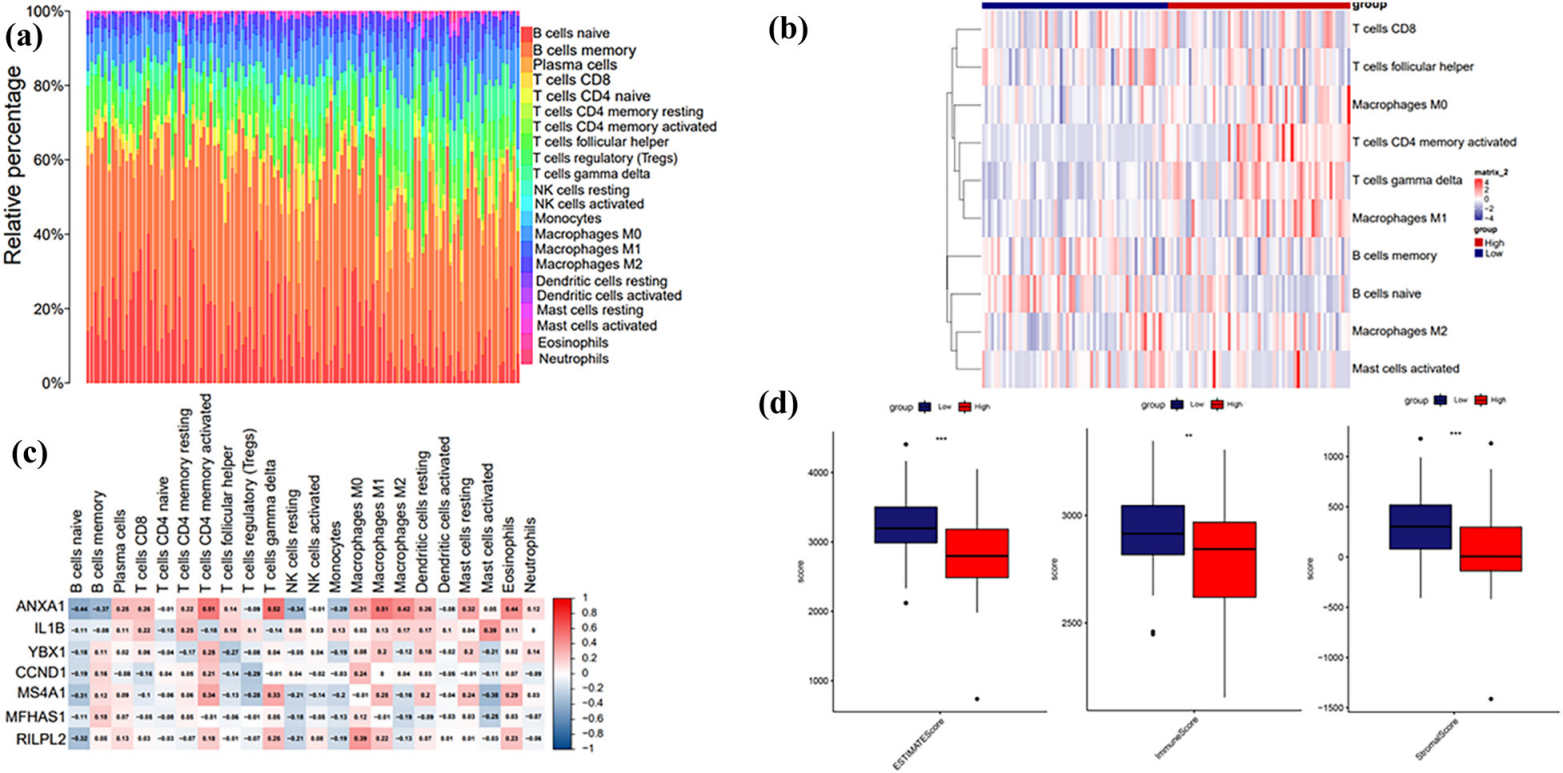
Tumor microenvironment and immune infiltration analysis. (a) Tumor microenvironment analysis of MCL; (b) analysis of immune infiltration in high and low risk groups; (c) correlation of immune cells and seven candidate genes; (d) ESTIMATE score, immune score, and stromal score for the high- and low-risk groups.

To analyze the immune cells content in MCL tumor microenvironment, we used CIBERSORT to assess the proportion of 22 immune cells in MCL tumor bed (Figure 6a). We were interested in the different cellular compositions in tumor microenvironment of high- and low-risk groups. As shown in Figure 6b, “T cells CD4 memory levels activated,” “T cells gamma delta,” and “macrophages M1” infiltrations were significantly higher in the high-risk group than in the low-risk group. Next we further assessed the expressions of identified seven model genes in immune cells (Figure 6c). Using the CIBERSORT package to assess the ESTIMATE score, immune score, and stromal score for the high-risk and low-risk groups, we found that those scores were significantly lower in high-risk group than those in low-risk group (Figure 6d). Such finding might indicate that the immune cells were highly repressed in high-risk group.

### 4.2 Assessment of drug responses in MCL

**Figure 7:**
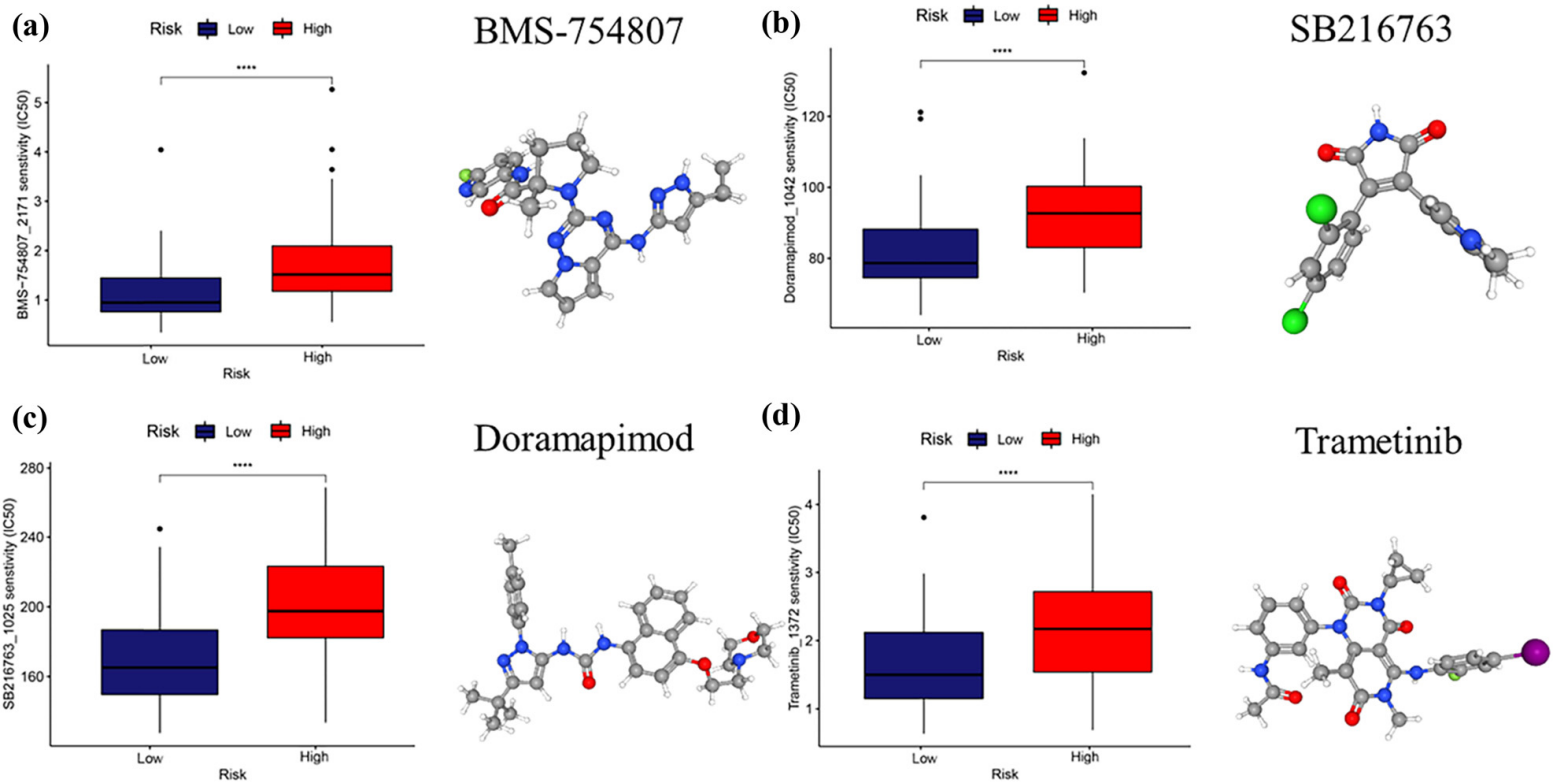
Drug responses analysis. (a)–(d) IC50 value and 3D structures of candidates’ drugs in high-risk and low-risk patients. Patients in the low-risk group were more sensitive to BMS-754807, SB216763, Doramapimod, and Trametinib.

The GDSC database contains cell line expression matrices and the half-maximal inhibitory concentrations (IC50) of the corresponding drugs. We used the oncoPredict package of R to assess the chemotherapy drug responses in high- and low-risk MCL groups. Figure 7a–d shows the four drugs, BMS-754807, SB216763, Doramapimod, and Trametinib, with the lowest *p* values; and the low-risk group MCL patients were more sensitive to those drugs than the high-risk group patients.

## 5 Discussion

In this study, we used MCL patients’ omics data, including single cell sequencing data and RNA sequencing data, to establish a ferroptosis-related prognostic model for MCL. We found that MCL patients could be stratified into two groups, high-risk group vs low-risk group, with the expressions of seven ferroptosis-related genes in the tumor cells. We also analyzed the immune cell composition in MCL tumors using the omics data, and showed the difference of cellular composition between high-risk and low-risk MCL patients. Finally, we compared the response to different chemotherapeutic agents between the high-risk group and low-risk group. We found that patients in the low-risk group were more sensitive to the four drugs BMS-754807, SB216763, Doramapimod, and Trametinib.

Previous studies have reported prognostic models of MCL, many of which were based on clinical factors. For example, the prognostic model constructed with six factors – age, physical fitness, white blood cell count, serum lactate dehydrogenase, bone marrow involvement, and serum albumin – was used to predict the outcome of MCL patients who received rituximab treatment [17]. The column chart developed by Zhu et al. utilized clinical data to predict OS of MCL [18]. In addition to prognostic models based on clinical factors, there were prognostic models constructed by tumor microenvironmental factors. In another prognostic model for MCL patients treated with rituximab, microenvironment components, such as T-reg cells, in lymph node biopsy were used to predict the patients’ survival [19]. Moreover, Hartmann et al. employed qRT-qPCR analysis of gene expression in MCL samples and introduced a novel survival predictor that outperformed the immunohistochemical marker Ki-67 [20]. Compared to those previous models, our prognostic model placed emphasis on the involvement of ferroptosis in MCL. The role of this iron-dependent form of cell death in tumors has garnered increased attentions, and may lead to the gradual development of ferroptosis-based immunotherapies.

In brief, ferroptosis is an iron-dependent, programmed cell death with lipid oxidation caused by ROS [3]. Intracellular iron ions catalyze the production of ROS via the Fenton reaction, a reaction that converts hydrogen peroxide into hydroxyl-free radicals. Subsequently, ROS cause lipid peroxidation, and ultimately induce cell death [21]. Iron is present in cells as balanced Fe^2+^ and Fe^3+^. Excessive intracellular Fe^2+^ catalyzes hydrogen peroxide to form hydroxyl radicals and hydroxides, and Fe^2+^ is also a cofactor mediating lipid peroxidation [22]. Since lipids are essential components of cell membrane, lipid peroxides, formed by free radical reactions with phospholipid polyunsaturated fatty acids, can induce cellular oxidative stress, and ultimately lead to cell damage [23]. Ferroptosis is a double-edged sword in tumorigenesis, having either promotional or inhibitory effects [24]. Dai et al. found that GPX4 depletion promoted pancreatic tumorigenesis, and ferroptosis inhibitor liproxstatin-1 suppressed the tumorigenesis [25]. Koppula et al. found that SLC7A11 overexpression in various cancers inhibited ferroptosis and ultimately promoted tumor growth, a result indicating ferroptosis was associated with tumor growth inhibition [26]. In addition, ferroptosis affected tumor invasion and immune cell infiltration. You et al. found that patients with high expression of ferroptosis-related genes in ovarian cancer had high immune cell infiltration in the tumor and high tumor aggressiveness [27]. In melanoma, ferroptosis inhibitor treatment increased the tumor cells’ metastasis [28]. CST1 stabilizes GPX4, inhibiting ferroptosis and promoting gastric cancer metastasis [29]. Finally, the activity of ferroptosis was related to the treatment outcome of different human cancers. Ferroptosis stimuli Erastin directly induced ferroptosis in tumor cells and increased the chemo-sensitivity of cancer cells to drugs [30]. In addition, ferroptosis stimuli might enhance the efficacy of radiation therapy [31]. Eventually, ferroptosis also affected the outcome of cancer immunotherapy by affecting immune cell activity [32].

Previous studies have shown that iron plays an important role in MCL cells. Aggressive MCL exhibited high iron metabolism [10]. Iron chelating agents could effectively induce MCL cells apoptosis [33]. Recently, Zhang et al. found that p53, the key suppressor gene of MCL, could negatively regulate the ferroptosis factor xCT. When the expression of p53 increased, there was a decrease in xCT, which reduced MCL ferroptosis [34]. However, the details of ferroptosis regulation in MCL, as well as the role of ferroptosis in MCL treatment, was still not clear. According to our model, the prognosis of MCL was correlated by ferroptosis-related, seven-gene expression signature. The seven genes were ANXA1, IL1B, YBX1, CCND1, MS4A1, MFHAS1, and RILPL2. ANXA1 encodes Annexin A1 protein, which regulates immune responses [35]. A previous study showed that a fragment peptide of ANXA1 could reduce ROS and lipid peroxides in mouse cardiomyocytes, thereby inhibiting ferroptosis and ultimately reducing lipopolysaccharide damage to cardiomyocytes [36]. Lack of ANXA1 in hairy cell leukemia resulted in tumor aggressiveness [37]. The second factor, IL1B was a pro-inflammatory cytokine with a role in T and B cells regulation. Downregulation of IL1B was detected in advanced stage of T-cell lymphoma [38]. A previous study suggested that IL1B was a ferroptosis-related diagnostic marker in osteoarthritis [39]. Fang et al. identified IL1B as a necessary link for cholesterol-induced ferroptosis [40]. The third molecule, YBX1, was a nucleic acid binding protein that regulated tumor cell proliferation and migration [41]. Knocking down YBX1 in DLBCL led to a decrease in tumor cell viability [42]. In colorectal cancer, YBX1 regulated intracellular ROS homeostasis by activating the NRF2 promoter; and such regulation might be related to ferroptosis [43]. YBX1 can maintain cellular glutathione level by stabilizing SLC7A11 and protect cells from oxidative stress-induced ferroptosis [44]. CCND1 was a key factor in cell cycle regulation, and aberrantly expressed in different human cancers [45]. CCND1 causes chromosome instability and cell cycle dysregulation, and MCL is characterized by (11,14) translocation leading to CCND1 overexpression in cells [46]. Tang et al. found that NOX4 was a source of ROS, and NOX4 expression correlated with CCND1 [47]. Recently, CCND1 has been identified as an ferroptosis hub gene associated with idiopathic pulmonary fibrosis [48]. MS4A1, also known as CD20, bound to a variety of B cell surface molecules and regulated B cells differentiation [49]. Anti-CD20 antibodies showed anti-cancer efficacy in different human cancers, including MCL [50]. A previous study suggested that MS4A1 regulated lipid metabolism, which might lead to ferroptosis [51]. MFHAS1, a GTP-binding protein, played a role in the AKT signaling and NF-κB pathways [52]. In Hodgkin’s lymphoma, MFHAS1 had high mutation rate, which indicated a tumorigenesis role of MFHAS1 [53]. The last factor RILPL2 had prognostic value in different human cancers, such as endometrial cancer [54]. The function of RILPL2 in ferroptosis is yet to be demonstrated.

It is well recognized that the progression of tumors is not solely determined by the intrinsic characteristics of the tumor cells, but also influenced by the dynamic interplay between the tumor and its microenvironment. Bernard and his colleagues discovered that the survival of MCL cells was contingent upon B cell receptors (BCRs), and BCR-targeting therapy induced apoptosis in MCL [55]. The level of CD4+ T cells was elevated in indolent MCL, compared to aggressive MCL, which had low CD4+ T cells; and a high CD4:CD8 ratio was correlated with superior OS [56]. By Cox and LASSO regression analysis, we used seven ferroptosis related genes to construct a prognostic model, which might be used to predict the MCL patients’ survival. Early patient stratification was a key for personalized therapy and a benefit to the patients. Based on omics data, we also analyzed the characteristics of MCL immune microenvironment. We found that high-risk and low-risk patients exhibited disparities in T cell activity. A previous study showed that stimulation of CD8+ T cells through cancer immunotherapy resulted in tumor cells lipid peroxides and ferroptosis [57]. Thus, our finding warranted further investigation of the T cells and MCL ferroptosis.

We identified four small molecular compounds with different response effects for the high- and low-risk groups. BMS-754807 was an inhibitor of both IGF-type 1 receptor and insulin receptor, and IGF-1R was highly expressed on MCL, thus BMS-754807 might be a potential target for MCL [55]. The study found that BMS-754807 may also act as an inhibitor of GSK3 [58]. Both BMS-754807 and SB216763 were GSK-3β inhibitors. GSK-3β mediated cell signaling was critical for ferroptosis regulation. In addition, GSK-3β was important in MYC-driven lymphomagenesis and inhibitors targeting GSK-3β played an inhibitory role in many lymphomas [59,60]. Doramapimod, a p38 MAPK inhibitor, played a role in T cell proliferation [61]. Then, ferroptosis was recently found to regulate the p38 MAPK pathway [62]. Inhibition of the p38 MAPK pathway has been shown to induce apoptosis of MCL cells; however, further investigation was required to determine whether Doramapimod could efficiently target MCL [63]. In addition to inhibit GSK-3, SB216763 might also alleviate iron accumulation and reduce ROS, thereby directly regulating ferroptosis [64]. However, whether SB216763 could regulate ferroptosis in MCL needed further study. Trametinib was a well-known MEK inhibitor, which may be a therapeutic target against tumors [65]. However, the role of these inhibitors in MCL is yet to be reported, and further studies and clinical trials are needed to validate them in the future.

There are several limitations to our study. First, our prognostic model, as well as immune microenvironment analysis, was based on public databases and had not been validated by patients’ samples. Second, we did not conduct any mechanistic study to investigate the role of those seven identified hub genes in MCL ferroptosis and prognosis. In addition, we did not examine the anti-MCL agents that we identified with wet-experiments. Third, the number of patients involved in this study was limited, in particular for validation cohort. Datasets containing more patients were needed to adjust the accuracy of our model.

To our knowledge, this was the first ferroptosis-related prognostic model of MCL. Since there were no publications addressing ferroptosis in MCL, our bioinformatics model at least indicated that ferroptosis was active in MCL. In our seven-gene prognostic model, each gene’s function in ferroptosis and in MCL was also unclear, and worthy for further investigation. We may then need to validate the viability of the prognostic model, particularly in terms of prediction accuracy, through long-term studies, and the accumulation of more patient data. Simultaneously, further studies are required to substantiate the safety and efficacy of the potential drugs identified in the study.

## 6 Conclusion

We constructed and validated a prognostic model with ferroptosis-related gene for MCL, including ANXA1, IL1B, YBX1, CCND1, MS4A1, MFHAS1, and RILPL2.

## Appendix

**Table A1:** Patient clinical characteristics of GSE42854

			Risk group		
Variable		Total cohort	Low-risk	High-risk	*p* value
Sex	Male	14 (66.67%)	7 (63.64%)	7 (70%)	1.000
	Female	7 (33.33%)	4 (36.36%)	3 (30%)	
Age	≤65	13 (61.91%)	7 (63.64%)	6 (60%)	1.000
	>65	8 (28.09%)	4 (36.36%)	4 (40%)	
CCND2 Translocation	Negative	9 (42.86%)	6 (54.55%)	3 (30%)	0.387
	Positive	12 (57.14%)	5 (45.45%)	7 (70%)	
Stages	I	1 (5.26%)	1 (10%)	0 (0%)	1.000
	II	3 (15.79%)	2 (20%)	1 (11.11%)	
	III	3 (15.79%)	1 (10%)	2 (22.22%)	
	IV	12 (63.16%)	6 (60%)	6 (66.67%)	
Treatment	R-CHOP	6 (33.33%)	3 (30%)	3 (37.5%)	0.171
Response	PR	3 (20%)	1 (11.11%)	2 (33.33%)	0.773
	CR	9 (60%)	6 (66.67%)	3 (50%)	
	PD	3 (20%)	2 (22.22%)	1 (16.67%)	
